# Differentially Expressed Genes in *Hirudo medicinalis* Ganglia after Acetyl-L-Carnitine Treatment

**DOI:** 10.1371/journal.pone.0053605

**Published:** 2013-01-04

**Authors:** Giuseppe Federighi, Monica Macchi, Rodolfo Bernardi, Rossana Scuri, Marcello Brunelli, Mauro Durante, Giovanna Traina

**Affiliations:** 1 Dipartimento di Ricerca Traslazionale e Delle Nuove Tecnologie in Medicina e Chirurgia, Università di Pisa, Pisa, Italy; 2 Dipartimento di Biologia, Università di Pisa, Pisa, Italy; 3 Dipartimento di Scienze Agrarie, Genetica Alimentari e Agro-Ambientali, Università di Pisa, Pisa, Italy; 4 Dipartimento di Scienze Economico-Estimative e degli Alimenti, Sezione di Chimica Bromatologica, Biochimica, Fisiologia e Nutrizione, Università degli Studi di Perugia, Perugia, Italy; National Eye Institute, United States of America

## Abstract

Acetyl-l-carnitine (ALC) is a naturally occurring substance that, when administered at supra-physiological concentration, is neuroprotective. It is involved in membrane stabilization and in enhancement of mitochondrial functions. It is a molecule of considerable interest for its clinical application in various neural disorders, including Alzheimer’s disease and painful neuropathies. ALC is known to improve the cognitive capability of aged animals chronically treated with the drug and, recently, it has been reported that it impairs forms of non-associative learning in the leech. In the present study the effects of ALC on gene expression have been analyzed in the leech *Hirudo medicinalis*. The suppression subtractive hybridisation methodology was used for the generation of subtracted cDNA libraries and the subsequent identification of differentially expressed transcripts in the leech nervous system after ALC treatment. The method detects differentially but also little expressed transcripts of genes whose sequence or identity is still unknown. We report that a single administration of ALC is able to modulate positively the expression of genes coding for functions that reveal a lasting effect of ALC on the invertebrate, and confirm the neuroprotective and neuromodulative role of the substance. In addition an important finding is the modulation of genes of vegetal origin. This might be considered an instance of ectosymbiotic mutualism.

## Introduction

Acetyl-l-carnitine (ALC) is the acetyl ester of the trimethylated amino acid L-carnitine that plays an essential role in energy production as a “shuttle” of long-chain fatty acids between the cytosol and the mitochondria for subsequent β-oxidation. ALC is involved in the control of ATP levels, the mitochondrial acyl-CoA/CoA ratio, peroxisomal oxidation of fatty acids, and mitochondrial enzyme activities. It is known that ALC has neuromodulatory, cytoprotective, antioxidant, neurotrophic, anti-apoptotic, and anti-aging effects, and, in addition, it improves the cognitive capability of aged animals chronically treated with ALC ([1]–[4], for a review see [5]). In previous studies, the effects of ALC on simple forms of nonassociative learning have been analyzed in the model of the swimming induction in the leech *Hirudo medicinalis* [6]. In particular, ALC reduces the sensitization process induced by brush strokes in a dose- and time-dependent manner, and provokes a reduction of the dishabituation [6]. In *H. medicinalis*, it is known that sensory stimulation initiates swimming activity by recruiting the mechanosensory neurons (T, P and N cells) which drive the flow of information to the swim-related muscles through a complex neuronal network [7]. Either T or N cell activity was affected by ALC: Lombardo et al. [8] reported that ALC increases the activation threshold of N cells, reducing the probability of activating them, and, in so doing, exerts an anti-nociceptive action. This effect is in accordance with studies in invertebrates demonstrating that ALC has analgesic properties and it is significantly effective in reducing neuropathic pain [9]–[12], although its mechanisms of action are not fully known. ALC also affects leech T cells, producing an increase in the amplitude of the afterhyperpolarization (AHP) which accompanies T cells bursting, by potentiating the Na^+^,K^+^-ATPase activity [13]. This modulation of the AHP amplitude leads to changes in the synaptic efficacy of T cells [13], [14], [15]. Since previous studies showed that a single treatment with ALC has persistent effects which increase in time [6], it is possible to hypothesize a modulation of gene expression. At present, there is evidence for a modulation of ALC-induced gene expression in the nervous system of rats chronically treated with the drug [16]–[22], but nothing is known about this in the leech. Therefore, in order to clarify whether a single ALC treatment is capable of regulating gene expression in *H. medicinalis*, suppression subtractive hybridisation (SSH) methodology has been used for generating subtracted cDNA libraries and subsequently identifying differentially expressed transcripts in the leech nervous system.

## Materials and Methods

### Animals

Adult leeches *H. medicinalis* (8–10 months old) purchased from Ricarimpex (Eysines, France) were utilized. The animals were kept in aquarium at 15–16°C, exposed to natural light/dark cycle. ALC or saline was supplied dorsally by two injections (one in the rostral and the other one in the caudal art of the body of the leech), each one of 100 µl/g animal as reported in Ristori et al. [6]. ALC was freshly prepared, dissolved in saline solution and, if needed, buffered to 7.4 pH with NaOH before use. Saline solution contained: 115 mM NaCl, 4 mM KCl, 1.8 mM CaCl2, 10 mM glucose, buffered to 7.4 pH by 10 mM Tris-maleate.

### Total RNA Isolation

A group of leeches has been injected with physiological solutions (control group, C), whereas another group with 2 mM ALC (Sigma Tau Laboratories, Pomezia Italy) (treated group, T). Eleven days after the treatment, total RNA was isolated according to Macchi et al. [23] and stored at −80°C until the RNA isolation.

### SSH Library Construction

Poly (A)+ RNAs were isolated from the pools of total RNAs of control and treated leeches using the *PolyATract*® *mRNA Isolation Systems (Promega, Madison, Wi, USA)* according to the protocol described by the manufacturer. Subtractive suppressive hybridisation (SSH) was performed according to Diatchenko et al. [24] using the *BD PCR-Select cDNA Subtraction Kit* (BD Biosciences) after the use of *BD SMART™ PCR cDNA Synthesis Kit* (BD Biosciences Clontech). The *SMART* procedure requires 0.025–1 µg of poly (A)+ mRNA to allow the amplification of the complete mRNA population contained in each sample (treated and control). The cDNAs from treated samples were respectively used as tester and driver for the *forward* and *reverse* subtraction according to the *BD PCR-Select cDNA Subtraction Kit*.

Amplified cDNA sequences from *forward* and *reverse* subtractions were directly inserted into a T/A cloning vector using the *TOPO TA Cloning Kit* (Invitrogen, USA) according to manufacturer’s instructions.

The efficiency of subtraction was evaluated by PCR amplification using the 5.8S ribosomal gene (the subtraction was efficient if the 5.8S transcript was reduced); the primers were designed on the 5.8S rRNA gene of *Xenopus laevis* (accession No. X02995.1) (Table 1).

**Table 1:** Primer sequences used in semi-quantitative RT-PCR assay.

Gene	Primer sequence	Accession No.
5.8S	F:CTTAGCGGTGGATCACTCGGCTCR: GCGACGCTCAGACAGGCGTAGC	X02995.1
Tubulin	F:CCAACCTGAATCGCTTGATTGGGR: GCTCAACATGCACACGGCACGC	U67677.1
Actinin	F: ACTCTTCAAACTAAATTGCGTCTCAGCR: GAATGGTCAACTCATTCTCGAGAGCC	HE962534
Hsp90	F: ATGTCACCAGGATGAAGGAGGGR: GCATATTCGTCAATAGCATCCACC	HE962533
Thiazole	F: TAATTGAGCAATCTGTGAGTCCTGGCR: GGAAGTAAAAAGAGCCGCGTGC	HE962538

### Differential Screening of the Subtracted cDNA Libraries

The obtained clones were cultured in LB medium with 10 mg/ml ampicillin in 96-well plates at 37°C. The cDNA fragments were amplified by PCR with nested PCR primers 1 and 2R, which were complementary to the adaptors, to check the presence and size of the individual fragments.

PCR reactions (25 µl) contained 18.5 µl sterile water, 0.6 µl of each primer (10 µM each), 2.5 µl 10× reaction buffer (Euroclone, Italy), 1 µl MgCl_2_ (Euroclone), 0.6 µl of dNTP mix (2.5 mM each), 0.25 µl Euro Taq polymerase (Euroclone) and 1 µl bacterial culture. Samples were denatured at 94°C for 10 min, followed by 30 cycles of 94°C for 30 sec, 68°C for 30 sec and 72°C for 1 min 30 sec, with a final extension at 72°C for 10 min. All PCR products were analyzed by agarose-gel electrophoresis. Two identical blots were created by spotting heated denatured PCR products (1 µl) from each of the clones of the subtractive libraries onto nylon membranes positively charged (Roche, Germany) and cross-linked by UV. The membranes were pre-hybridized for 3 h in prehybridization buffer (DIG easy Hyb, Roche), then incubated overnight at 42°C in hybridization buffer (DIG easy Hyb containing the labelled cDNAs obtained by DIG-DNA Labelling Kit Nonradioactive, Roche). To eliminate the background due to the presence of the adapter sequences (BD PCR-Select cDNA Subtraction Kit; BD Biosciences) in the probes, a high concentration (3 µg/ml) of oligonucleotides corresponding to the nested primers and the complementary sequences (competitors) previously used for the libraries construction have been added into the prehybridisation and hybridisation solution. Moreover, the adaptors have been removed by digesting cDNAs with RsaI restriction enzyme prior to probe labeling. The fragments that hybridized only with the labeled forward cDNA or that showed at least higher signals than the signals obtained with the reverse labeled cDNA were subjected to sequencing analysis.

### Clone Sequencing and Analysis

Differentially expressed clones were sequenced by automated sequencing (MWG Biotech Ebersberg, Germany). Homology searches of all sequences were compared to the GenBank EMBL-EBI database using the BLAST algorithm.

### Semi Quantitative Relative RT-PCR

Reverse transcriptions were carried out with total RNA (4 µg), previously treated with DNase I (Roche), isolated from ALC treated and control leeches with *SuperScript™ II RNase H- Reverse Transcriptase kit* (Invitrogen) and *Oligo(dT)12–18 Primer* (Invitrogen) according to the manufacturer’s instructions.

1 µl of first-strand cDNAs were used for each PCR amplifications and PCRs were performed using gene-specific primers and 5.8S rRNA gene of *Xenopus laevis* (accession No. X02995) or alpha-1 tubulin of *Hirudo medicinalis* (accession No. U67677.1) were used as housekeeping genes. Primer sequences are reported in Table 1.

The relative amounts of each PCR product were readily quantified by direct scanning with a densitometer of ethidium bromide-stained 2% agarose gel electrophoresis with Quantity One® Software (Bio-Rad, USA). To standardize the total RNA and the efficiency of cDNA synthesis of the samples, the band intensities were standardized with the average intensity of the 5.8S or Tubulin product across the samples investigated. The ratio between the value of the analyzed gene product level and the 5.8S or Tubulin product level of each sample was calculated from three independent experiments performed for each gene. The statistical analysis was done with the Unpaired T test (*GraphPad Prism 4.00 software*). All data are expressed as mean values ± SE.

## Results

The aim of this research was to single out genes that are differentially expressed in the leech nervous system in response to a single ALC treatment. In order to detect the differentially expressed genes, two groups of leeches were used: the first group was subjected to a single administration of 2 mM ALC and the second one was subjected to a single administration of saline solution. Eleven days after the treatment, chains of ganglia from both groups of animals were extracted, and the total RNA was isolated. We performed the suppression subtractive hybridization (SSH) method [24] for the construction of two subtractive cDNA libraries: the forward and reverse libraries consisting of transcripts positively and negatively modulated, respectively, by the treatment with ALC. The efficiency of subtraction was evaluated by PCR amplification of the housekeeping gene for 5.8S rRNA. The fragments are detectable as faint bands in the subtracted sample only after PCR amplification, while they are clearly detectable in the unsubtracted sample.

About 400 cDNA clones for each cDNA library were collected and the clones showing a single band after PCR amplification were sequenced. About 70% of the analyzed cDNA clones from all libraries resulted differentially expressed. We sequenced 40 differentially expressed cDNA clones, belonging to different cDNA libraries. With the information gathered from different databases we identified and assigned a putative function to more than half of the sequenced clones (Tables 2–3). Sequences believed to be interesting from a physiological point of view are presented in Table 2: we report also some *alien* sequences in Table 3 that pose some interesting questions that will be analysed in the discussion.

**Table 2:** Identification of up-regulated cDNA sequences from CNS of *Hirudo medicinalis* treated with ALC.

Clone	Accession No^(a)^	Accession No. of matching Sequence^(b)^	Putative identification^(c)^	Biological process^(d)^
IB9	HE962518	ABF54967	Ribosomal protein P1	Plays an important role in the elongation step of *protein* synthesis
IC9	HE962519	EAW88342.1	Udp-N-acteylglucosamine pyrophosphorylase 1-like	Catalysis of the transfer of a nucleotidyl group to a reactant.
IA10	HE962520	XP_002266398	Histidine decarboxylase-like	Cellular amino acid metabolic process
1AA3	HE962521	CBM42048	Heat shock protein-70 kDa	Response to stress
1AA7	HE962522	O13224	Heat shock protein hsp20	Response to stress
10AA3	HE962539		EST	
10AA4	HE962523	XP_003617296	Alanine glyoxylate aminotransferase	Pyrimidine base metabolic/nucleoside catabolic process
1AB77AH3IB67AC2	HE962524HE962526HE962527HE962525	XP_002331152 XP_002331152XP_002331152XP_002331152	Hypothetical protein	
1AB8	HE962529	AAS87603.1	Cytochrome P450	Oxidation-reduction process
1AC2	HE962530	ABP01769.1	Non-specific lipid transfer protein	Lipid transport
1AF5	HE962531	AAC25158.1	Retinoic acid converting enzyme	Retinol metabolism,
1AF6	HE962533	ABB29612	Heat shock protein 90 kDa	Response to stress
7AH2	HE962532	ACJ86240	Rhodanese homology domain protein	Domain found in the phosphatases, dehydrogenases and stress proteins.
1AE6	HE962534	NP_001072666	Actinin	Important structural and regulatory roles in cytoskeleton organization and muscle contraction
10AH8	HE962528	XP_003637605	Hypothetical protein	

a Accession number of the clones;

b Accession number of the best match sequence;

c Identification based on sequence similarity;

d Biological process according to http://www.uniprot.org/.

**Table 3:** Identification of up-regulated cDNA sequences of vegetal origin from CNS of *Hirudo medicinalis* treated with ALC.

Clone	Accession No.^(a)^	Accession No. ofmatching Sequence^(b)^	Putative identification^(c)^	Biological process^(d)^
1AA10	HE962535	AEX60843	Ribulose-1,5-bisphosphate carboxylase/oxygenase small subunit	Photosynthesis/Photorespiration
1AB67AG3	HE962536HE962537	AAP35043.1AAP35043.1	Chlorophyll a/b binding protein	Photosynthesis, light harvesting
1AE5	HE962538	ADG27845	Thiazole biosynthetic enzyme	Thiamine biosynthetic process

a Accession number of the clones;

b Accession number of the best match sequence;

c Identification based on sequence similarity;

d Biological process according to http://www.uniprot.org/.

**Figure 1:**
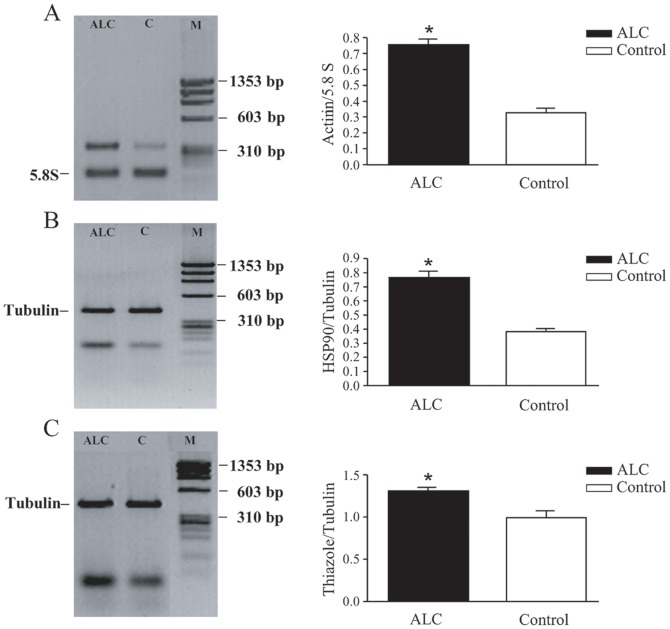
Semiquantitative analysis. Relative RT-PCR (left) of Actinin (A), HSP90 (B) and Thiazole biosynthetic enzyme (C) transcripts. The relative expression levels (right) were calculated as ratio of each analysed transcripts with respect to the Tubulin or 5.8S product levels. The values followed by different letters are significantly different at the 0.05 probability level according to Unpaired T test. ALC and C, treated and control leeches respectively. M, molecular weight marker.

As shown in Tables 2–3 1AE6, 1AF6 and 1AE5 clones code for Actinin, HPS90 and Thiazole biosynthetic enzyme respectively. Surprisingly the last clone codes for a protein, the Thiazole biosynthetic enzyme, which is generally expressed only in plants. The proteins Actinin and HPS90 are involved at different levels in neuronal activity. Because we previously observed that a single treatment with ALC in the leech affects forms of non associative learning, (6), here we thought it would be interesting to point out whether the treatment with ALC modulates the expression of the genes coding for these proteins. The technique of relative RT-PCR was used to analyze the expression of the 1AE6, 1AF6 and 1AE5 clones. The results obtained showed that ALC positively modulates the expression of genes coding for: Actinin (ALC 0.757±0.034, Control 0.326±0.029; n = 3; t = 9.645, df = 4, p = 0.0006; Fig.1A), HSP90 (ALC 0.766±0.043, Control 0.383±0.021; n = 3; t = 8.004, df = 4, p = 0.0013; Fig. 1B) and Thiazole biosynthetic enzyme (ALC 1.310±0.040, Control 0.991±0.081; n = 3; t = 3.531, df = 4, p = 0.0242; Fig. 1C).

## Discussion

Studies on the effects of ALC on nonassociative learning forms in *H. medicinalis* showed that ALC is able to produce a long-lasting effects on sensitization, dishabituation, and habituation processes [6]. In particular, a single administration of ALC brings about long-term effects that occur even 11 days after the treatment. The fact that ALC has long lasting effects suggested that it could lead to a modulation of gene expression and, consequently, a differential mRNA synthesis in the leech nervous system. This hypothesis was evaluated with a molecular approach using the technique of Suppression Subtractive Hybridisation (SSH). SSH permits the construction of cDNA libraries containing differentially expressed transcripts in a tissue in response to a treatment, or to a different physiological condition. The analysis of expression of the clones considered has shown that ALC modulates the expression of several genes: among them, the genes coding for Actinin, HSP90 protein and the biosynthetic enzyme for Thiazole were chosen for semi-quantitative analysis. The interest in these clones lies in the fact that some of they code for proteins involved in neuromodulatory and neuroprotective mechanisms and the last is expressed normally in plant cells and this arises interesting considerations.

Actinin is a ubiquitous cytoskeletal protein identified in many eukaryotic organisms, such as humans, mice, *Drosophyla melanogaster* and *Caenorhabditis elegans* [25]–[28]. It is classified in four different major isoforms [29]. Man, and probably other vertebrates, express all four isoforms, while invertebrates and protists have only one isoform. The members of the α-Actinin family are very represented in the mammals CNS, in particular, in dendritic spines. There is evidence that α-Actinin is involved in the modulation of glutamatergic receptors [30], [31]: α-Actinin is associated with both AMPA and NMDA receptors (NMDAR) [32]–[35] whose importance in the mechanisms of synaptic plasticity and neuronal development is well known, and it represents a key component of the macromolecular complex mediating the calcium-dependent inactivation of NMDAR. In the leech, there is evidence that sensory neurons such as tactile T neurons, trigger glutamatergic polysynaptic connections activating NMDAR-dependent mechanisms linked to activity-dependent synaptic plasticity and behavioural processes [36], [37]. Our data show an ALC-induced up-regulation of the expression of the gene for α-Actinin suggesting a mechanism through which ALC might reduce the responsiveness to tactile stimuli in the behavioral performances that have previously been shown to be impaired by ALC treatment [6]. A modulation of the expression of the gene coding for α-Actinin during learning has also been observed in the rat. Experiments carried out to identify genes involved in synaptic plasticity during fear conditioning, have shown that after 30 minutes and up to 4 hours of conditioning, there is an increase in α-Actinin expression [38].

The heat shock proteins (HSPs) are a class of proteins produced in response to various stress conditions [39]. HSPs prevent the inappropriate aggregation of proteins and mediate the transport of immature proteins to the target organelles [40]. Therefore they are essential for cell survival because they help the correct folding and refolding of new proteins or the degradation of proteins damaged by stress. HSP90 is one of the most abundant cytosolic proteins in eukaryotes, even in absence of stress [41]. This protein is a *chaperone*, acting towards certain classes of proteins, such as receptors for steroid hormones and protein kinases, and it may contribute to protein homeostasis in physiological conditions and stress [42]. Interestingly, Gass et al. [43] demonstrated that HSP90 protein is constitutively expressed in neuronal cells of the adult rat brain and Blackshaw et al. [44] found an HSP90 from selected clones from a 24-h regenerating ganglion subtracted library just in *H. medicinalis* thus suggesting a functional role of HSP90 in the physiological molecular program of neurons. In 2004, Gerges et al. [45] identified the role of HSP90 in excitatory synaptic transmission in the hippocampus, observing that this *chaperone* is necessary for efficient neurotransmitter release. HSP90 is an important component of the molecular machinery required for continuous cycling of AMPA type glutamate receptors. These results highlight the importance of HSP90 as a regulator of synaptic processes [45]. In the leech nervous system there is evidence of glutamatergic transmission at the level of sensory integration [35]. Our findings suggest the hypothesis that HSP90 modulation might influence synaptic control during plastic changes.

The biosynthetic enzyme for Thiazole is an enzyme involved in the biosynthesis of thiamin (vitamin B1) [46], necessary in the metabolism of proteins, carbohydrates and fats. Among its different functions, the coenzyme thiamine is also important in the synthesis of acetylcholine, glutamate and GABA [47] and its deficiency can play an important role in some neurodegenerative disorders [48], [49], [50] such as Alzheimer’s disease and Wernicke encephalopathy [51]. Finally, thiamine, and other B group vitamins seem to have important antinociceptive and anti-inflammatory effects [52]–[55]. therefore they are useful for the treatment of certain pain conditions, such as low back pain, or trigeminal neuralgia [52], [53]. ALC increases the expression of the gene coding for the biosynthetic enzyme for Thiazole, and this effect might be linked to the anti-nociceptive action of the drug.

The troubling problem that arises is that the biosynthesis of thiamin is normally codified in plant genomes but not in the animal genome.

As far as the finding of *alien* plant genes is concerned, it must be stressed the fact that several plant genes are found in different animal systems. In the comparative genome analysis of planarian ESTs, Mineta et al. [56] found that about 30% of planarian nervous system-related genes had homologous sequences in *Arabidopsis* and yeast. The authors speculate that during evolution many genes, that are functional in the nervous system or CNS, may have been recruited from genes used in unicellular systems. It is worth noting that in our system the alien sequences are present as clones, but the corresponding genes were not found in leech DNA (unpublished results).

Korneev et al. [57] have constructed a subtractive cDNA library from regenerating Retzius cells of the leech: among the up-regulated sequences during nerve regeneration was found a hypothetical 39.4 yeast protein.

Blackshaw et al. [44] in their studies of the molecular basis of nervous system repair in *H. medicinalis* nerve cells found two clones coding for a heat shock protein HSP90 and a 16S ribosomal RNA as those reported in Table 2 of this paper. Moreover, they found a clone coding for an *Arabidopsis thaliana* ubiquitin-conjugating enzyme (UCE) and another coding for the cytochrome oxidase subunit II of the red alga *Cyanidium caldarium*: interestingly, these were up-regulated at 24 h post-axotomy.

One can hypothesize a symbiotic relationship with unicellular algae. This type of interactions is well known in different systems (for a review see Venn et al. [58]). More recently, an association was found between embryos of the salamander *Ambystoma maculatum* and the green alga *Oophila amblystomatis* [59]. The authors considered this association as an instance of ectosymbiontic mutualism.

Aljamali et al. [60] in their transcriptome analysis of *Amblyomma americanum* salivary glands found a sequence of *Pisum sativum* root nodule estensin. Moreover, one conting of the library was highly similar to *Lens culinaris* nonspecific lipid transfer protein 1 precursor (LTP1). The authors hypothesized the possible involvement in a nonspecific uptake of arachidonic acid. It worth noting that in our library is present a LTP2 sequence.

A recent interesting and questioned paper by Zhang et al. [61] reported evidence of cross-kingdom regulation by exogenous plant microRNA in the sera and tissues of various animals. According to the authors, plant miRNAs are primarily acquired orally, through food intake.

In conclusion, the data collected in this paper reveal a lasting effect of ALC which modulates gene expression. Although our results do not fully explain the effects previously observed in behaviour, we show that a single administration of ALC is able to affect gene expression in the nervous system of the leech *H. medicinalis*. Previously, we have detected a modulation of gene expression by ALC in the rat brain [17]–[22]. By comparing the genome libraries obtained in both animal models we did not find common genes whose expression has been modulated by ALC except some HSPs. This difference in ALC-modulated gene expression might depend on different treatment modalities (single administration in the leech and chronic treatment in the rat). Also the time at which the analyses have been performed (11 days after the single treatment in the leech and immediately after the treatment in the rat) might account for the difference observed in gene expression as well as the phylogenetic distance between the animal models.

Nevertheless, the use of SSH has allowed to construct cDNA library in *H. medicinalis* and to identify differential expressed genes in response to a specific pharmacological treatment, thus representing a good tool for future investigations in the molecular biology of the leech nervous system.
